# Relationship among surface electric double layer of cardiomyocyte membrane and toxicology of digoxin and opening of ion channels

**DOI:** 10.1038/s41598-022-25205-2

**Published:** 2022-12-01

**Authors:** Ying Zhou, Yanfei Hao, Pei Sun, Ming Chen, Ting Zhang, Hong Wu

**Affiliations:** 1 The Sixth Medical Center, Chinese People’s Liberation Army General Hospital, Beijing, 100142 China; 2 The Eighth Medical Center, Chinese People’s Liberation Army General Hospital, Beijing, 100091 China; 3 The Medical Security Center, Chinese People’s Liberation Army General Hospital, Beijing, 100039 China

**Keywords:** Chemical biology, Physiology, Cardiology

## Abstract

We applied a new idea that the potential effect can change the ion adsorption structure on the cell surface to explore the mechanism of digoxin poisoning and the regulation of ion channels. The effects of digoxin on the electrophoretic mobility and behaviors (non-contraction or contraction or autorhythmicity) of cardiomyocytes were observed by single-cell electrophoresis technique (imitate the opening method of in vivo channel) and the method of decomposing surface potential components on the cells. As well as affect the association with electrical activity. The results suggested that the increase of cardiomyocytes transmembrane potential and the Na^+^–K^+^ exchange on the cell surface of the action potential phase 4 caused by the poisoning dose of digoxin, leading to the oscillation of adsorbed ions on the cell surface and the incomplete channel structure, which were the mechanism of cardiac ectopic beats. The results revealed that the opening of ion channels is regulated by the surface electric double layer of the cell membrane.

## Introduction

Our previous work systematically studied the surface potential and electromotive potential of cardiomyocytes, and understood the composition of the charged components on the surface of cardiomyocytes (i.e., plasma membrane (lipid bilayer and membrane protein), surface glycoconjugates and transmembrane potential), as well as the characteristics and interrelationship of each component^1^. It was found that during the electrical activity of cardiomyocytes, the inflow of ions comes from the adsorbed ions on the cell surface^2^; the change of cell surface potential can regulate the opening of ion channels by affecting the surface electric double layer on the cell. In recent years, the research on colloidal interface involves many fields such as energy, materials, biology, etc., among them, a single electric double layer has been widely used to describe the charge distribution at the interface, as well as to study the electrical properties of non-uniform fixed charge distribution membranes by network simulations^3,4^. However, the surface structure of cardiomyocytes is very complex, and its electromotive process is accompanied by its own electrical activity, and the distribution of surface charges cannot be explained by a single electric double layer. The theory of ion migration on cardiomyocytes requires new models and new ideas, that is, the result of the synergistic effect of multiple electric double layers. In order to prove the new idea, we constructed an electrodiffusion model of the ventricular myocyte interface, using the effect of digoxin on the living cardiomyocyte membrane under different conditions, as a comparison of the model, combined with the various effects of digoxin on the electromotive process and electrical activity, to verify the fit between the two.

Digoxin is a drug of the digitalis family for the treatment of heart failure, and it is also one of the main tools used to study the mechanism of arrhythmia^5^. Pharmacological mechanism is clear and associated with electrical activity of ventricular myocytes^6,7^. It selectively binds to Na^+^, K^+^-ATPase at the plasma membrane level and inhibits enzyme activity, which increases intracellular Ca^2+^ concentration and enhances myocardial contractility. But at the same time, digoxin also increases intracellular Na^+^ concentration, decreases K^+^ concentration, and increases extracellular K^+^ concentration^8,9^. During treatment, the occurrence of severe arrhythmia is the main cause of death due to digoxin poisoning. The main clinical manifestations include atrial, junctional, ventricular; fast or slow; single or continuous ectopic beats and conduction block, the mechanism is still not very clear^10–12^.

The results showed that the changes in the electrophoretic mobility (EPM) and the behavior of the cardiomyocytes in the model and our new idea, namely, the ion migration mode on the cardiomyocytes surface has reached a high degree of fit; Such results well reveal the toxicology of digoxin and gating mechanism of ion channels.

This study includes the following: a brief description of the electrodiffusion model at the ventricular myocyte interface; An experimental study exploring the effects of digoxin on EPM and behaviors of ventricular myocytes using single-cell electrophoresis techniques and methods to decompose cell surface potentials (mainly discussing cardiac ectopic beats); As well as make analysis and reasoning about the conclusions of the research.

## An electrodiffusion model of the ventricular myocyte interface

Based on the membrane structure and electrical activity characteristics of ventricular myocytes, we briefly describe the model and the electrodiffusion-adsorption process. The plasma membrane layer (lipid bilayer and membrane protein) of ventricular myocytes membrane is 50 nm away from the surface glycoconjugates^13^, which is much larger than the thickness of two electric double layers. Thus, there are two inner-outer parallel electric double layers on the cell surface, which are divided into the inner electric double layer of the membrane and the surface electric double layer of the membrane (Supplementary Fig. 1). The two electric double layers are relatively independent and related to each other, and the transmembrane potential connects the surface potentials into a whole by its spatial effect, and exert unequal effects on the two electric double layers. Among them, the gating-associated protein of the Na^+^ channel that initiate electrical activity is located in the surface electric double layer of the membrane, and the outward K^+^ channel ends at the outer surface of the membrane. While the exchange of Na^+^–Ca^2+^ and Na^+^–K^+^ in the electrical activity is carried out in Ψ_mi_ and the adsorption layers to which it belongs. The two parallel electric double layers are the structural basis for the formation of the action potential platform and the phase 4 plasma membrane layer ion exchange (Supplementary Fig. 2). The electrical activity process and the electromotive process of the cardiomyocytes are linked together, and the phases 0–3 of the action potential are completed in the external electric field. The formation of the electric double layer is completely different from ordinary colloidal particles, its repolarization is accomplished in two manners. In cell depolarization, the Na^+^ and Ca^2+^ of inflow come from the surface adsorption layer of the membrane and the inner electric double layer of the membrane, the cell repolarizes, and the outflow of K^+^ return here^2^. The two manners include: K^+^ outflow to the level of surface glycoconjugates (next, the flow direction of ions: outside → inside) and ion exchange at the plasma membrane level (amount of 3Na^+^–1Ca^2+^ exchange is less than 3Na^+^–2K^+^ exchange)^14^, the exchange process generates ion current, direction: inside → outside (two ion currents are convective). Among them, the former is completed at the end of the phase 3 of the action potential, the surface electric double layer of the cell membrane is restored intact (the total electrochemical potential equilibrium inside and outside the cell is restored), and the repolarization process generates a transmembrane potential (mainly due to the outflow of K^+^ and the exchange of 3Na^+^–2K^+^); The latter until the phase 4^12^ (at this time, the external electric field has disappeared). Due to the hysteresis, the inner electric double layer of the membrane is still separated (Na^+^–K^+^ and Na^+^–Ca^2+^ exchange can continue). The outward ion current generated by the exchange is bound to affect the two electric double layers on the cell surface. This will cause some of the adsorbed ions on the surface diffusion layer of the cell membrane to return to the suspension and/or change the thickness of the electric double layer (to maintain the total electrochemical potential equilibrium inside and outside the cell). This effect on the surface diffusion layer (EPM) of the cell membrane mainly depends the size of the outward ion current generated by the exchange and the original (unaffected) surface diffusion layer of the cell membrane (as well as the transmembrane potential and the two electric double layers). So above, the adsorbed ions on the cell surface form a unique distribution.

Cardiomyocyte EPM studies showed that under the selected experimental conditions, the cells EPM were non-linear oscillations^1^, and the ζ _ms_ potential and Ψ _ms_ potential were not positively correlated. For example again, when the transmembrane potential was from − 96.8 to 0 mV (suspension K^+^ concentration was 3–140 mM), the cell EPM trend was: − 96.8 to − 49 mV (3–20 mM K^+^ concentration), which is gradually increased with oscillation (Supplementary Fig. 3a); at − 49 to 0 mV (20–140 mM K^+^ concentration), which oscillates in a high-amplitude region (Supplementary Fig. 3b). At the same time, the cardiomyocytes showed irregular alternation of the three behaviors (Supplementary Fig. 3c). The effect of ion concentration on cell EPM can be found in^1,2^.

The results suggested that under clinically visible conditions (10–3 mM K^+^ concentration) and the structure of two parallel electric double layers, the two repolarization manners together with the synergistic effect of the gradual increase of the transmembrane potential, have the effect of reducing the cell EPM (i.e., making surface diffusion layer of the membrane smaller and thinner). More adsorbed ions are distributed to the surface adsorption layer of the membrane and the inner electric double layer of the membrane. Especially the ion exchange of the plasma membrane layer of the action potential phase 4 (influence of unidirectional ion flow will be obvious).

## Results

Cardiomyocytes showed different features under an electric field depending on the experimental conditions. Although the cells may contract under some suspension (extracellular fluid) conditions or may not, the impact of the contraction on the electrophoretic speed did not affect the analysis and judgment of experimental results measured from different conditions. Commonly, cardiomyocytes showed negative charges in the electric field. But their electrophoretic speed may have obvious difference even in a same suspension, suggesting that their surface potential components have different composition ratios (for example, it is also related to where the cells come from the ventricular wall). In fact, cells were possibly affected by the difference in time of enzyme action, mechanical damage, and so on in the preparation. However, this also did not impact on the judgment of variation tendency of the cell mobility.

### Impact of digoxin on EPM and behavior of the cells

**Figure 1:**
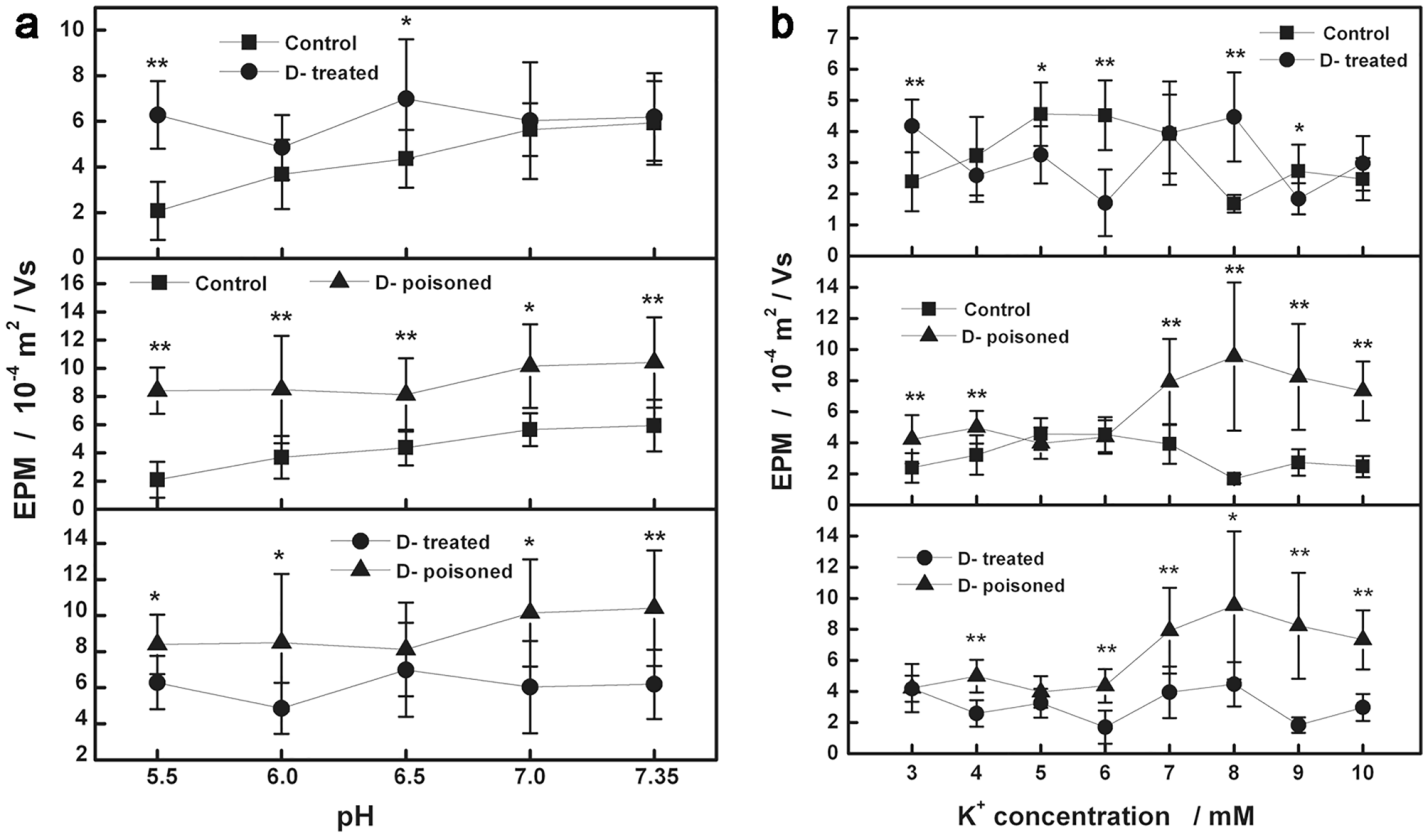
In different suspensions impact of digoxin on EMP of cardiomyocyte. (**a**) Changes of three groups EPM measured in the suspensions of 145 mM NaCl, 2 mM CaCl_2_, 4 mM KCl and pH 5.5–7.35. (**b**) Three groups EPM measured in the suspensions of 145 mM NaCl, 2 mM CaCl_2_, 3–10 mM KCl and pH 7.35.

In different pH suspensions, the cardiomyocyte EPM data of the three groups showed that the mean value of EPM ranged from 2.08 to 5.93 (10^–4^ m^2^/Vs) for the control group, from 4.86 to 6.99 (10^–4^ m^2^/Vs) for the treated group; and from 8.12 to 10.41 (10^–4^ m^2^/Vs) for the digoxin poisoned group. The differences between the groups were illustrated in Fig. 1a. For the behavior of cardiomyocytes, in the case of external electric field free, the observed number of autorhythmic cells was 4 in the control group, 8 in the treatment group, and 31 in the poisoning group. After applying an external electric field, the number of non-contraction cells was 29 in the control group (data related to pH), 16 in the treatment group, and 18 in the poisoning group; while the number of contraction cells was 27 in the control group, 36 in the treatment group, and 11 in the poisoning group (Table 1).

**Table 1:** The behavior of cardiomyocytes in the experiment (n = 12).

Cell suspension condition	Control			Treatment			Poisoning		
	Non-contraction	Contraction	Auto-rhythmicity	Non-contraction	Contraction	Auto-rhythmicity	Non-contraction	Contraction	Auto-rhythmicity
**Different pH**									
5.5	12	0	0	5	5	2	4	3	5
6	6	5	1	4	5	3	5	2	5
6.5	5	6	1	4	7	1	3	1	8
7	3	8	1	1	10	1	3	3	6
7.35	3	8	1	2	9	1	3	2	7
Total	29	27	4	16	36	8	18	11	**31**
**Different K**^**+**^									
3 mM	2	8	2	4	6	2	0	8	4
4 mM	2	8	2	4	3	5	6	1	5
5 mM	2	3	1	2	7	3	5	2	5
6 mM	3	2	7	7	0	5	5	2	5
7 mM	5	6	7	4	5	3	8	2	2
8 mM	1	7	4	1	7	4	4	1	7
9 mM	1	7	4	3	4	5	8	1	3
10 mM	2	3	7	5	3	4	5	3	4
Total	*18	44	*34	30*	35	**31	41*	20*	**35
**140 mM K**^**+**^									
5.5	1	6	5	0	2	10	0	1	11
6	2	6	4	0	4	8	6	1	5
6.5	1	5	6	0	3	9	2	0	10
7	0	0	12	0	3	9	1	0	11
7.35	0	0	12	0	0	12	5	0	7
Total	*4	17	*39	*0	12	**48	14	*2	**44

A single symbol denote P < 0.05, double symbol denote P < 0.001.

The symbol on the left of the total number is the comparison of the control group with different pH conditions and other groups; the symbol on the right side of the total number is the comparison of the treatment group and the poisoning group with the control group under different conditions.

When the K^+^ concentration changed (3–10 mM) in the suspension, the mean EPM of the control group was in the range of 1.68–4.56 (10^–4^ m^2^/Vs); the mean value of the treatment group was 1.71–4.47 (10^–4^ m^2^/Vs); the mean value of the poisoning group was 3.97–9.54 (10^–4^ m^2^/Vs). Differences between the groups were shown in Fig. 1b. When no external electric field was applied, the behaviors of cardiomyocytes showed that the number of autorhythmic cells was 34 in the control group, 31 in the treatment group, and 35 in the poisoning group. After applying an external electric field, the number of non-contraction cells was 18 in the control group, 30 in the treatment group, and 41 in the poisoning group; the number of contraction cells was 44 in the control group, 35 in the treatment group, and 20 in the poisoning group (Table 1).

### After cell surface potential decomposition

**Figure 2:**
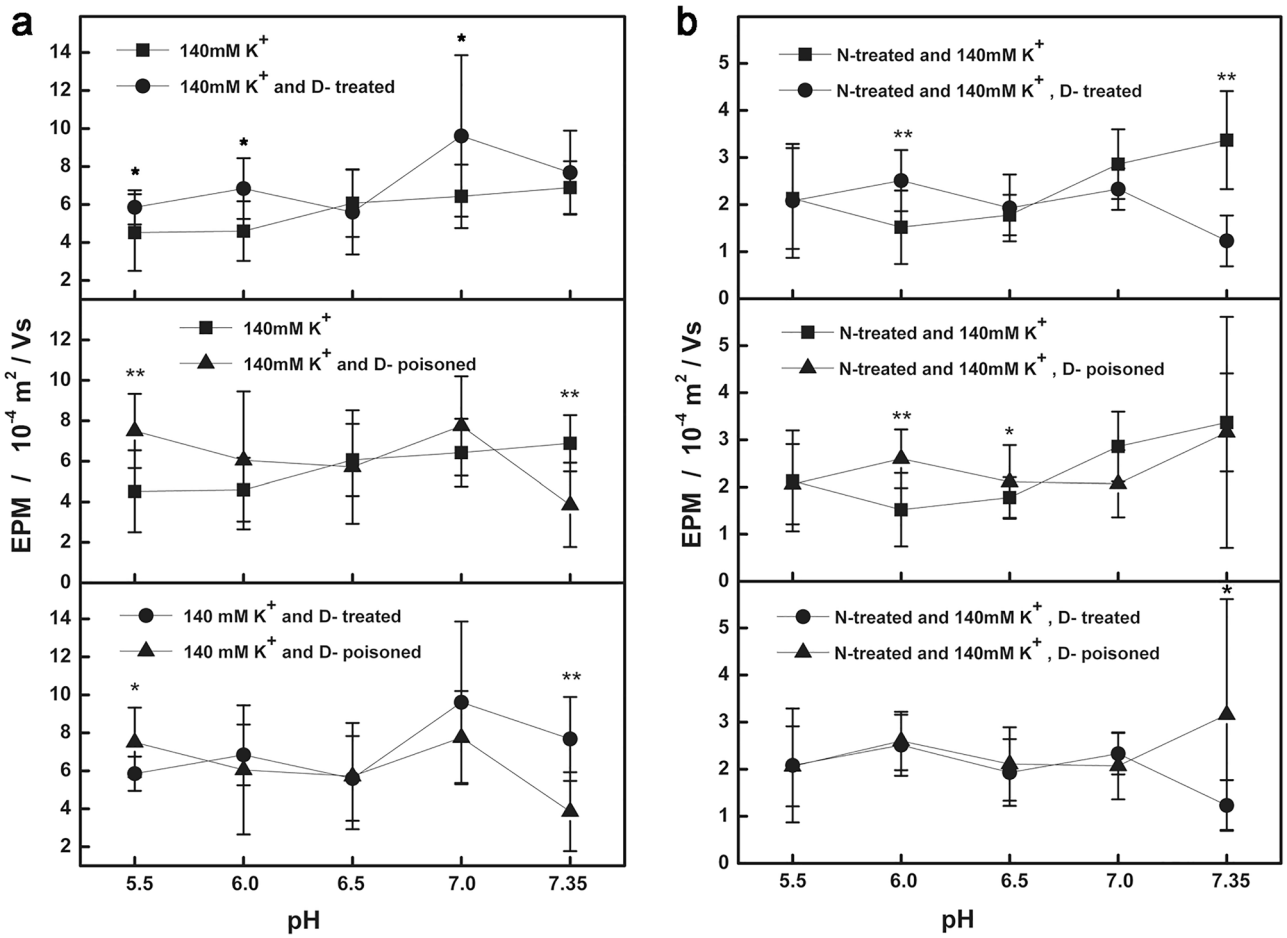
After decomposition of surface potential impact of digoxin on EMP of cardiomyocyte. (**a**) Three groups EPM of cardiomyocytes measured in the suspensions of 140 mM KCl (transmembrane potential was zero), 10 mM NaCl, 2 mM CaCl_2_ and pH 5.5–7.35. (**b**) After the cardiomyocytes are treated with neuraminidase, three groups EPM measured in the suspensions of 140 mM KCl (transmembrane potential was zero), 10 mM NaCl, 2 mM CaCl_2_ and pH 5.5–7.35.

When the suspension was 140 mM K^+^ (the transmembrane potential was zero), the mean EPM of the control group was in the range of 4.52–6.89 (10^–4^ m^2^/Vs); the mean EPM of the treatment group was 5.6–9.61 (10^–4^ m^2^/Vs); and the mean EPM of the poisoning group was 3.85–7.75 (10^–4^ m^2^/Vs). The differences between the groups were shown in Fig. 2a. When no external electric field was applied, the behaviors of cardiomyocytes showed that the number of autorhythmic cells was 39 in the control group, 48 in the treatment group, and 44 in the poisoning group. After applying an external electric field, the number of non-contraction cells was 4 in the control group, 0 in the treatment group, and 14 in the poisoning group; the number of contraction cells was 17 in the control group, 12 in the treatment group, and 2 in the poisoning group (Table 1).

**Figure 3:**
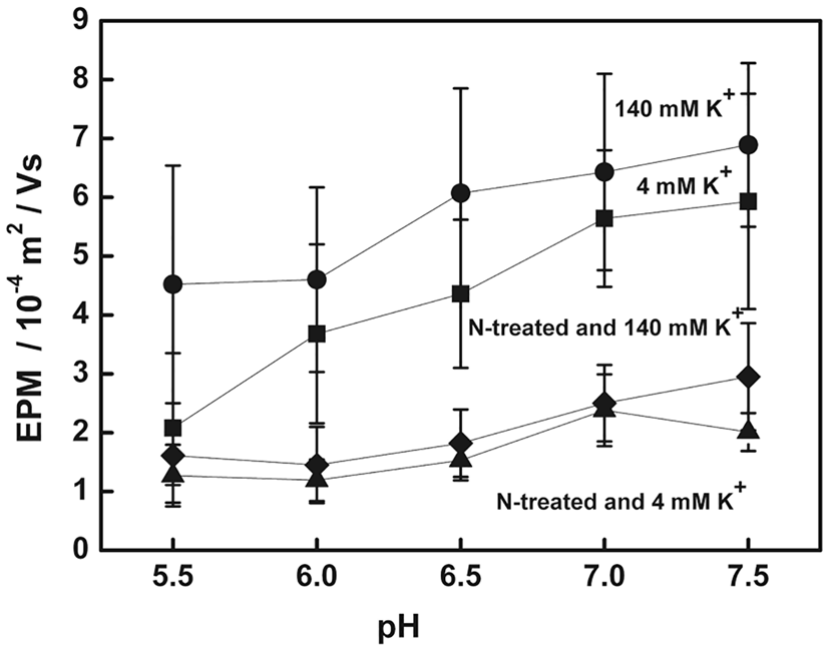
Impact of cellular surface potential components for the EPM of cardiomyocyte. 4 mM K^+^: Suspension in 145 mM NaCl, 2 mM CaCl_2_, 4 mM KCl and pH 5.5–7.35. 140 mM K^+^: Suspensions in 140 mM KCl (transmembrane potential was zero), 10 mM NaCl, 2 mM CaCl_2_ and pH 5.5–7.35. N-treated and 4 mM K^+^: After treatment with neuraminidase, suspensions in 145 mM NaCl, 2 mM CaCl_2_, 4 mM KCl and pH 5.5–7.35. N-treated and 140 mMK^+^: After treatment with neuraminidase, and suspensions in 140 mM KCl (transmembrane potential was zero), 10 mMNaCl, 2 mM CaCl_2_ and pH 5.5–7.35.

After the cells were treated with neuraminidase and suspended in a solution of 140 mM K^+^, which removed the influence of surface glycoconjugates and transmembrane potential, the mean EPM of the control group was in the range of 1.22–5.07 (10^–4^ m^2^/Vs); the mean EPM of the treatment group was 1.32–2.51 (10^–4^ m^2^/Vs); the mean EPM of the poisoning group was 2.06–3.41 (10^–4^ m^2^/Vs). The differences between the groups were shown in Fig. 2b. After the surface glycoconjugates and transmembrane potential were removed, none of the cardiomyocytes contracted under the selected suspension conditions. While removing the cellular surface glycoconjugates, but retaining the transmembrane potential (suspension conditions: 145 mM NaCl, 2 mM CaCl_2_, 4 mM KCl, pH 7.35), the mean EPM of the group (n = 12) was in the range of 1.19–2.38 (10^–4^ m^2^/Vs) (Fig. 3); the behavior showed that the number of autorhythmic cells was 0, the number of non-contraction cells was 5, and the number of contraction cells was 7.

**Figure 4:**
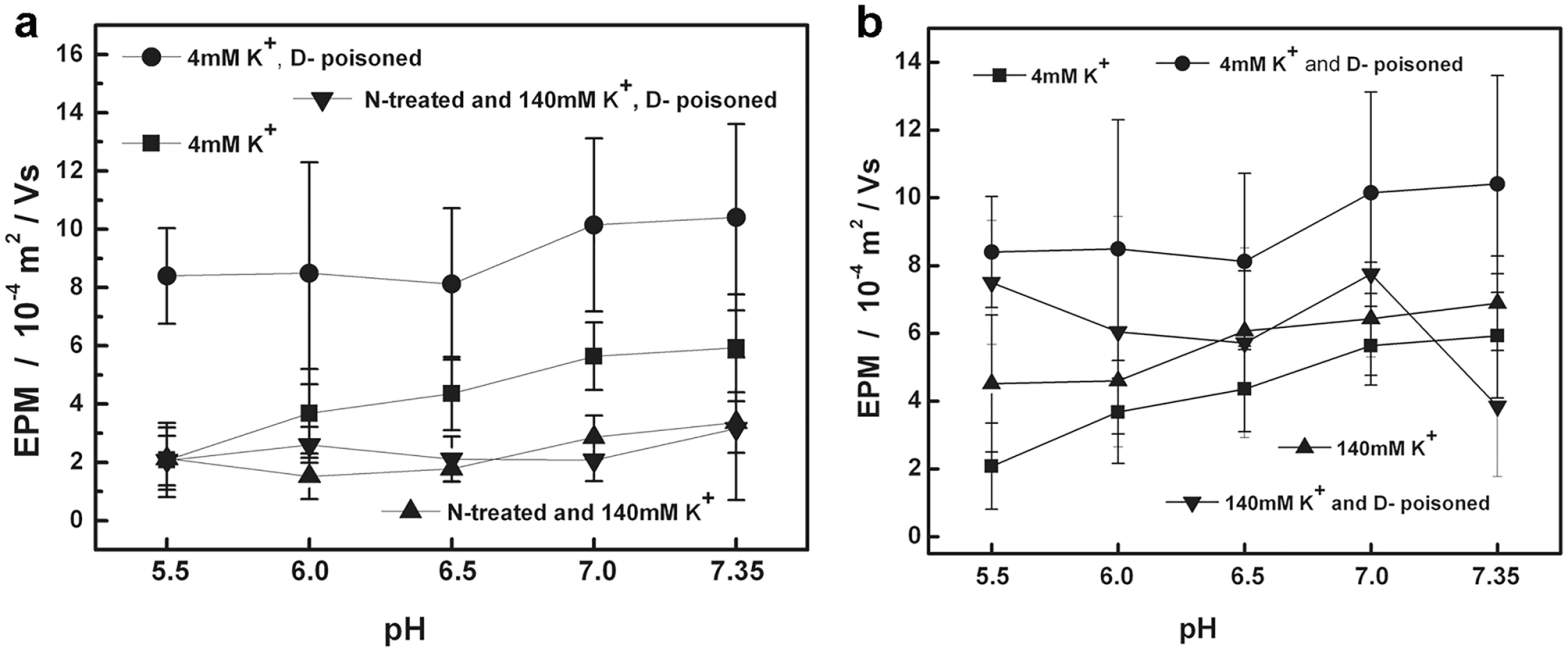
Comparison of the effects of digoxin poisoning dose on cardiomyocytes EPM with different potential components. The annotations are shown in Figs. 1a, 2a and b.

It is obvious that different doses of digoxin had different effects on the EPM and behavior of cardiomyocytes under different conditions before and after surface potential decomposition. For the EPM data recombination, that is, the cardiomyocytes were in the following conditions: suspended in a solution close to physiological conditions (4 mM K^+^ concentration); suspended in a solution that removed the transmembrane potential (140 mM K^+^ concentration); and after removing surface glycoconjugates, re-suspended in the above two solutions, the changes in cell EPM were compared to obtain Fig. 3. Figure 4 was a comparison of the effect of digoxin poisoning dose on cell EPM before and after surface potential decomposition.

In addition, the results showed that the increase of the external electric field strength not only increased the amplitude of the cell EPM; within a certain strength range, the number of cells with contraction behavior will also be increased.

## Discussion

Generally, to trigger the beating of cardiomyocytes (effective whole cell contraction), two conditions are mainly required: a specific and sufficient number of inflow ions (that is, changes in ionic concentration and ratio in the intracellular fluid → intracellular Ca^2+^ release) and Ca^2+^ in the intracellular fluid increase to a certain concentration. Between them, the increase of intracellular Ca^2+^ concentration depends on inflow of ions (and ion inflow requires opening the ion channel first)^14^. Therefore, the cardiac ectopic beats caused by digoxin are mainly related to the abnormal opening of ion channels.

EPM can reflect the amount of ions lost on the cell surface (in the external electric field, a shear plane is formed on the cell surface); and indirectly reflect the characteristics of the surface potential change of cells under different experimental conditions^15^. In studies related to the electrical activity of cardiomyocytes, the reason for using single-cell electrophoresis technology is because the changes in the surface electric double layer of cardiomyocytes membrane in an external electric field are the same as the initiation of the electrical activity of cardiomyocytes in vivo (the external electric field in the body comes from sinoatrial node cells or charged cells around or pacemakers)^14,16^. In general, the opening of gated ion channels in cardiomyocytes involves two elements: the presence of an external electric field or changes in transmembrane potential; and a change in the state of the channel (closed to open). Among them, the change of channel state is the key to opening the channel. Application of an external electric field (such as in vivo) and changing the transmembrane potential (such as patch clamp experiments) act on the gating-associated proteins in different ways to achieve the same effect of opening the channel. So, what are the mechanisms of opening ion channels in two different ways; what role does the cardiomyocyte EPM (or diffusion layer) play in it?

### Difference between digoxin dose and EPM

The therapeutic dose of digoxin appears to have a selective effect on the cardiomyocytes EPM with intact surface potential. Under different pH conditions, digoxin can increase the cellular EPM, but the increase amplitude is different. At different K^+^ concentrations, the EPM values of cardiomyocytes oscillated around the EPM curve of the control group, and in the suspensions with 3, 5, 6, 8 and 9 mM K^+^ concentrations, the EPM differences between the two groups were statistically significant. The results showed that although the therapeutic dose of digoxin could cause changes in the cardiomyocyte EPM curve in the control group, the cardiomyocyte EPM did not increase significantly in suspensions close to physiological conditions. This should be related to the small changes in ions in their intracellular and extracellular fluids.

Under different pH conditions, the toxic dose of digoxin had a significant effect on the EPM of cardiomyocytes, and the EPM of the cells was significantly increased. The difference between most poisoning group and treatment group was statistically significant. Under different K^+^ conditions, most K^+^ concentrations (except 5 and 6 mM K^+^ concentrations) in the suspension showed a significant increase in the EPM of cells, especially at 7–10 mM K^+^ concentrations, the difference was statistically significant. The increase in cell EPM was more pronounced with increasing K^+^ concentration in suspension (above physiological range). It is shown that the toxic dose of digoxin can increase the cardiomyocytes EPM, and digoxin poisoning and high K^+^ concentration may have a synergistic effect on the increase of cell EPM.

### Difference in surface potential and EPM

In order to further understand the effect of digoxin on the surface potential of cardiomyocytes, we used a method of decomposing cell surface potential under the premise of ensuring cell survival. Due to the distance between the plasma membrane layer and the surface glycoconjugates is 50 nm, which is much larger than the usual thickness of the electric double layer^17,18^. Theoretically, when the concentration of extracellular K^+^ is 140 mM, the theoretical value of the cell transmembrane potential is zero. At this time, the measured cell EPM is more likely to reflect the cell mobility associated with the surface glycoconjugates. The therapeutic dose and toxic dose of digoxin have a certain effect on EPM associated with glycoconjugates on the cell surface, which is raised or lowered and appears to be related to pH. After the cell is treated with neuraminidase, the surface glycoconjugates is removed, and the concentration of 140 mM K^+^ in the suspension, only the charge of the plasma membrane layer remained on the cell surface, this is where the Na^+^, K^+^-ATPase is located. Results showed that the therapeutic dose and toxic dose of digoxin had a small effect on the cell EPM associated with the plasma membrane layer, and it was not strong. Since cardiac glycosides bind to the Na^+^, K^+^-ATPase in a 1: 1 ratio^19^, with high affinity and selectivity, it is speculated that the number of Na^+^, K^+^-ATPase^20^, which may bind to digoxin, is limited compared with the total negative charge of the plasma membrane. However, this result showed that the effect of digoxin on cell EPM was closely related to the surface structure of the cell membrane.

Figure 3 showed the effect on cell EPM after the surface potential components of cardiomyocytes were gradually removed. At 4 mM K^+^, it is the cardiomyocyte EPM with intact surface potential. When the transmembrane potential of cardiomyocytes was removed (140 mM K^+^ concentration), the cell EPM is raised. And if the surface glycoconjugates was removed, the transmembrane potential was removed, or not, the EPM amplitude is reduced to minimum, and the difference between the two is small, showing the difference in cell EPM between single component and multicomponent coexistence. That is, the effect of the transmembrane potential on the cell EPM was restricted by the surface structure (two parallel electric double layers). Figure 4 showed the effect of digoxin poisoning dose on the EPM of cardiomyocytes with three surface structures (intact surface potential, potential with only plasma membrane, equivalent to potential with only surface glycocomplexes). The results further showed that the EPM of cardiomyocytes with a single electrical double layer (Fig. 4a) and two parallel electrical double layers (Fig. 4b) responded differently to digoxin poisoning, even when the transmembrane potential was zero, the cell EPM only reflected the potential associated with surface glycocomplexes, cell EPM still showed high amplitude changes.

### Digoxin, surface potential and behaviors

The three behaviors of cardiomyocytes were distributed differently among the three groups (control group, digoxin treatment group, and digoxin poisoning group) under different conditions. Since both high K^+^ and digoxin can lead to changes in transmembrane potential, we selected control groups with different pH conditions as the reference objects for each group (4 mM K^+^ concentration in this group). This group had the fewest autorhythmicity cells (6.7% of the three behaviors in this group), and the difference in the number of contracting and non-contracting cells was smaller. Compared with it, the number of autonomic cells in the three groups was highest in the digoxin treatment and the digoxin poisoning group at a concentration of 140 mM K^+^ (80% and 73.3% of the three behaviors in the groups, respectively); while under high K^+^ conditions, the group with the least autorhythmicity cells was the digoxin treatment group with different K^+^ concentrations (32.2% of the three behaviors in the group). However, if the group data (7, 8, 9 and 10 mM K^+^) in this group far beyond physiological K^+^ concentrations were removed, the number of autorhythmicity cells decreased significantly. The results showed that the autorhythmicity cells were more concentrated in the high K^+^ concentration group, the digoxin poisoning group and the coexistence of the above two conditions (and the high K^+^ concentration combined with digoxin treatment group), compared with the control group under different pH conditions, the difference was statistically significant (Table 1). Combined with the changes in cell EPM, it was found that the autorhythmicity cells in the high K^+^ concentration and digoxin poisoning group (also seen in the treatment group) were partially accompanied by an increase in EPM (some significantly increased). It was shown that the increase in the number of autorhythmicity cells is related to changes in the surface potential of cardiomyocytes (transmembrane potential and other potential components).

Furthermore, when only the glycoconjugates on the cell surface were removed, under physiological suspension conditions (4 mM K^+^ concentration), the cells still displayed three behaviors, showing that the cell surface channel structure can be reorganized^21^. And simultaneous removal of surface glycoconjugates and transmembrane potential, cardiomyocytes will not contract (and no autorhythmicity), which is different from cardiomyocytes with intact surface potential. The main reason is that the cell surface potential and the electrochemical potential inside and outside the cell are a whole. Removing the two and applying an external electric field will greatly change the electrochemical potential inside and outside the cell (at this time, the Na^+^ concentration in the suspension is only 10 mM), which will affect the ion inflow (or change the direction of ion inflow). The relationship between cell EPM, contraction behavior and external electric field strength is the same as in electrophysiological experiments, and a certain strength of external electric field is required to trigger electrical activity. These also indicate that the opening of ion channels is related to the loss of adsorbed ions in the electric double layer on the surface of the cell membrane, that is, the importance of the electric double layer on the cardiomyocytes surface for the channel structure.

### Cellular electrophysiological toxicity associated with digitalis membrane toxicity

For the electrophysiology of ventricular myocytes, the toxicity of digitalis mainly includes: (1) The Na^+^, K^+^-ATPase of cells is inhibited, resulting in a concomitant change in the ion concentration inside and outside the cell. Among them, the change of K^+^ concentration (intracellular decrease, extracellular increase) caused the increase of cell transmembrane potential (i.e., negative value decreases)^18,22^. When the K^+^ concentration in the suspension is 7–10 mM, the theoretical value of the transmembrane potential is − 75.5 to − 66.5 mV, while the gating voltage of Na^+^ channel is − 70 to − 60 mV (transmembrane potential)^14^. (2) The K^+^ conductance of the cell membrane is increased, there is a difference between the maximum diastolic potential of the cell and the K^+^ equilibrium potential and the slope of phase 4 depolarization is increased. Miura and Rosen^5,23^, in response to these signs of high K^+^ concentration on the cell surface speculated that ‘there was an accumulation of potassium at the exterior surface of the cell membrane’. Obviously, this does not conform to the principle of ion adsorption and exchange on the membrane surface. During the repolarization process, the outflow of K^+^ comes to the surface adsorption layer of the membrane and the inner electric double layer the membrane, making K^+^ accumulate here. In the phase 4 of the action potential, the external electric field disappears and surface diffusion layer the membrane returns. There is bound to be an exchange of Na^+^ and K^+^ on the cell surface (and associated exchange) to comply with the principle that the composition of adsorbed ions on the surface obeys the extracellular fluid. The exchange will bring the above-mentioned K^+^ situation and delayed afterdepolarizations (or with ectopic beating)^5^. Delayed afterdepolarizations are caused by pacing at the early stage of Na^+^–K^+^ exchange on the cell surface, and have similar reasons as the refractory period of the cell to stimulation (i.e., a certain amount of adsorbed Na^+^ is required to initiate electrical activity, which is the source of inflow Na^+^). The presence or absence of Na^+^–K^+^ exchange on the cell surface is also the difference between the digoxin poisoning and the zero transmembrane potential or high K^+^ in suspension (although their transmembrane potential both increased), the latter has no Na^+^–K^+^ exchange on the cell surface.

Toxic doses of digoxin increase the resting transmembrane potential of cardiomyocytes, which can keep the Inflow ion channel (such as Na^+^ channel) on the cardiomyocytes surface in an open state, resulting in continuous electrical activity (single or continuous cell contraction); toxic doses of digoxin inhibits Na^+^–K^+^ exchange at the plasma membrane layer can result in a cell surface Na^+^–K^+^ exchange in phase 4 of the action potential, leading to a single cell contraction and delayed afterdepolarizations (i.e., a brief disruption of electrical activity). These toxic manifestations of cardiomyocytes are the basis for the formation of clinical malignant arrhythmias.

The electrophysiological toxicity of digitalis in cardiomyocytes is highly consistent with the reasons for the changes in cellular EPM and behavior caused by digoxin toxic doses in this experiment. The study of electrokinetic phenomena in cardiomyocytes complements the multiple effects of digitalis toxicity on cells, elucidating the toxicology of digoxin (and digitalis).

### Regulation of ion channels

**Figure 5:**
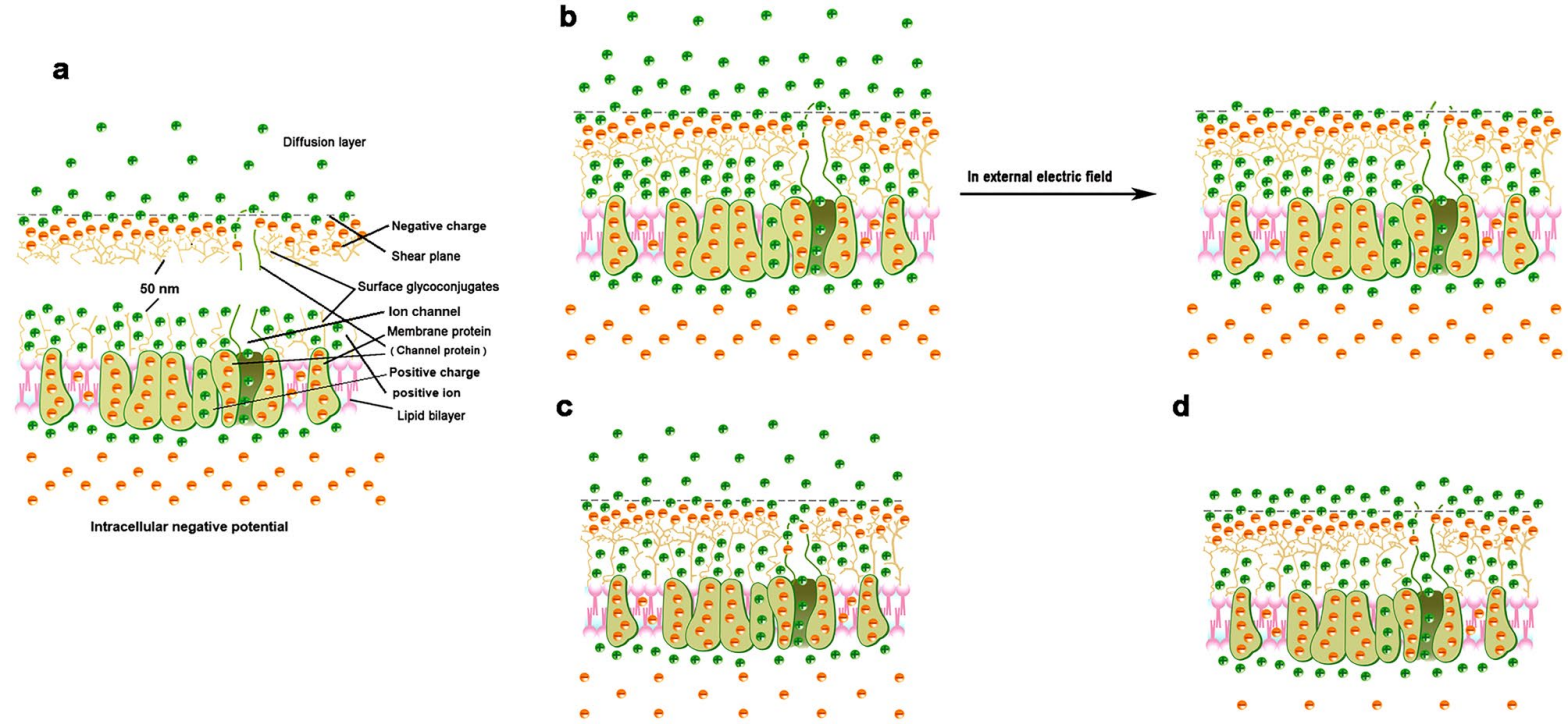
Influence of potential effect on the ion adsorption structure of the cardiomyocytes surface. Schematic diagrams of the surface structure of cardiomyocytes (**a**). Under physiological conditions, when an external electric field is applied (or in a negative electric field), the surface structure of the cardiomyocytes membrane changes (**b**); the K^+^ channel (outward) of cardiomyocytes is similar to it. When the transmembrane potential changes (within a certain range), the surface electric double layer on the cell membrane changes (thickness and diffusion layer), and cardiomyocytes can show three behaviors: contraction (**b**), or non-contraction (**c**), or autorhythmicity (**d**).

In terms of the unique surface structure of cardiomyocytes and the properties of colloidal particles, the application of an external electric field and the change of the transmembrane potential first affected the surface electric double layer of the cell membrane. Therefore, whether the cell was in an external electric field, the surface electric double layer of cell membrane was separated and negatively charged; or increasing the transmembrane potential caused the negative potential on the cell surface to decrease, all of which will reduce the number of adsorbed ions on the cell surface and make the surface electric double layer of the cell membrane smaller and thinner. For the channel gating-associated protein in the membrane protein, the negative charge it carries forms a binding relationship with some adsorbed ions on the surface electric double layer (that is, the complete structure of the channel includes the adsorbed ions associated with it). When the number of adsorbed ions decreases (and the surface electric double layer becomes thinner) to, the ions bound by the channel-gating associated proteins return to the solution, it will cause structural (and conformational) changes in ion channels and destroy the integrity of the closed ion channel^24–28^, leading to channel opening (or arrhythmia) (Fig. 5). If there are no ions bound to channel gating-associated proteins in the adsorbed ions of the returned solution, it will not initiate electrical activity and no cell contraction^29–31^. Similarly, if the ions bound to the gating-associated protein compete with the ion channel blocker for exchange (or combination) and cannot be dissociated in the electric field, the ion channel will be blocked^32–34^. In addition, if the EPM amplitude is too high (on the premise that the surface potential becomes small), that is, the ions in the cell surface diffusion layer are excessive, these ions are not tightly bound to the charges, which exacerbates the instability of ion channels.

In a nutshell, normally, the location of ions bound with the channel gating-associated protein in the surface electric double layer of the cell membrane, which is the key to opening the ion channel (the condition for opening the channel is that the channel structure becomes incomplete). Changes in the transmembrane potential can alter the shape of the electric double layer and the location of “bound ions” in the surface electric double layer. This is why changes in transmembrane potential can cause ion channels to open^35^; the external electric field takes away the bound ions of channel gating-associated proteins by separating the electric double layer on the cell surface, which is the mechanism of gated Na^+^ channel opening in vivo; the depolarization process, the adsorbed ions on the cell surface (including the bound ions of the gating-associated protein) flow into the cell sequentially, showing that different ion channels are opened sequentially. And the significant increase in transmembrane potential and the Na^+^–K^+^ exchange on the cell surface of the action potential phase 4 caused by digoxin poisoning are the causes of cardiac ectopic beats. The adsorbed ions on the cell surface are an important part of the cell surface structure^16,36,37^, and the size and shape of the surface electric double layer of the cell (or membrane) are equally important, which depends on the multi-component surface potential of the cell and the composition and changes of the ions in the suspension. For example, chemical-gated channels: chemical substances compete with ions and bind to membrane proteins, which makes the surface electric double layer on the cell smaller and thinner; intracellular sarcoplasmic reticulum Ca^2+^ channel: ions flow into the cell, changes the ion concentration of intracellular fluid, resulting in deformation of the electric double layer of the sarcoplasmic reticulum surface (because the electric double layer of the sarcoplasmic reticulum surface has only one ion adsorption layer—the influence of the transmembrane potential, and the characteristics of the ionic composition in this layer, the deformation is more easily induced by the change of Ca^2+^ concentration in the suspension). These all can open ion channels (Fig. 5d)^38–42^.

The experimental results and analysis are consistent with clinical findings. For example, digoxin poisoning can cause a variety of severe arrhythmias and even death^43^; When the concentration of K^+^ in suspension changes (greater or less than the physiological range), the cardiac autorhythmicity increases (arrhythmia occurs)^44,45^, which can also prepare cardioplegic solution, etc. (K^+^ concentration is greater than the physiological range)^46,47^. These also show that the K^+^ concentration (transmembrane potential) changes linearly, and the changes in EPM and cells behavior were non-linear.

The essence of the electrophoretic phenomenon and the characteristics of ion adsorption-diffusion of cardiomyocyte membranes, as well as a comprehensive understanding of the electrical activity of cardiomyocytes, have helped us understand and discover the relationship between ion channel opening and the surface electric double layer of the membrane. That is, the separation of channel gating-associated protein and its adsorbed ions, leading to incomplete channel structure is the key to opening ion channels and generating heartbeat (physiological and pathological). This research suggests that living body materials, complete structures and imitation of in-body conditions are very important for the study of life activity mechanisms.

## Methods

### Cell preparation

All animal experiments were carried out according to the guidelines in Directive 2010/63/EU of the European Parliament on the protection of animals used for scientific purposes; all experimental protocols were approved by the Committee on the Use and Care of Animals of the People’s Liberation Army General Hospital. Animal studies are reported in compliance with the ARRIVE 2.0 guidelines. In the implementation, the animals were anaesthetized with 4–5% isoflurane-O_2_ (inhalation) and killed by cervical dislocation.

Ventricular cardiac myocytes were isolated from adult Sprague–Dawley rats (2–3 months old, weight 225–300 g) using standard enzymatic techniques, as described previously^48^. Freshly isolated single cells were stored in Tyrode’s solution containing 137 mM NaCl, 5.4 mM KCl, 1.2 mM MgCl_2_, 1 mM NaH_2_PO_4_, 1 mM CaCl_2_, 20 mM glucose, and 20 mM HEPES (pH 7.4).

### Preparation of cell suspensions

Suspension 1: 145 mM NaCl, 2 mM CaCl_2_, 4 mM KCl, 10 mM glucose, and 20 mM HEPSE at pH 5.5, 6.0, 6.5, 7.0 and 7.35, respectively. Suspension 2: 145 mM NaCl, 2 mM CaCl_2_, 3–10 mM KCl (by Nernst equation of E_K_ = (− RT/F) In ([K]_i_/[K]_o_ to calculate the theoretical value of the transmembrane potential^12^), 10 mM glucose, and 20 mM HEPSE, pH 7.35. Suspension 3: 140 mM KCl, 10 mM NaCl, 2 mM CaCl_2_, 10 mM glucose, and 20 mM HEPSE at pH 5.5, 6.0, 6.5, 7.0 and 7.35, respectively. All the buffering solutions were adjusted to a viscosity at 3.8 mPa·s (24 °C) by addition of 0.5 mM hydroxypropyl methyl cellulose (HPMC).

### Removal of sialic acid from cardiomyocytes

Cardiomyocytes were suspended in Tyrode’s solution containing 0.25 U mL^−1^ neuraminidase (Sigma-Aldrich, St. Louis, MO, USA) at pH 6.0 (hematocrit at 0.5% v/v). After continually shaken at 37 °C for 90 min, the cells were washed with the same Tyrode’s solution for 3 times.

### Procedure

Cardiomyocytes were equally divided into three groups corresponding to control, treatment and poisoning group. They were separately washed three times with the experiment solution and then resuspended in the same solution at 0.1% (v/v) cells and kept at 24 °C. Enzyme-treated cardiomyocytes were suspended in the Suspension 3 or 1. Each group of the suspended cells was loaded on a microscopic cell electrophoresis system (Beijing Warder Biomedicine Instrument Company, China) and electrophoresed under an electric field strength at 5 V cm^−1^ in 2 min (in addition, the control group tried an electric field strength of 1–10 V cm^−1^). Only the living cells with similar mass and good state (texture clear of the membrane) were random recorded (instrument has measures to reduce electroosmosis). Each sample solution was determined repeatedly for 12 times. All the measurements were finished in 10 h after the isolation of cardiomyocytes. The same experimental conditions, three groups used 1-time isolated cardiomyocytes. In the experiments, we simultaneously observed the behaviors of cardiomyocytes: non-contraction (i.e., neither cell contracted when an external electric field was applied or not), contraction (cells contracted when an external electric field was applied) and autorhythmicity (no external electric field is applied, the cells are also contracting). Throughout the experiment, 80% of cardiomyocytes exhibited good appearance and viability.

### Drug

Digoxin (Sigma-Aldrich, St. Louis, MO, USA) stock solutions were prepared in 75% ethanol; the final concentration of ethanol did not exceed 0.03%^49^. The applied dose was 1.3 × 10^–6^ mM for the treatment group and 3.8 × 10^–6^ mM for the poisoning group^50^.

### Statistical analysis

Data was expressed as means and standard deviation. Group comparisons of EPM data were analyzed by two independent samples t-test and rank sum test. Group comparisons of behavioral data were analyzed by paired design (or unpaired) t-test (GraphPad Prism 6). Values with P < 0.05 were considered statistically significant. A single symbol denote P < 0.05, double symbol denote P < 0.001 as indicated.

## Supplementary Information

Supplementary Figures.

## Data Availability

The data supporting the findings of this study are available within the paper and its supplementary information file.

## References

[1] Zhou Y, Liu J, Wang J, Wang Y, Sun P (2009). Measurement of electrophoretic mobility of cardiomyocytes. Electrophoresis.

[2] Zhou Y, Hao Y, Sun P, Li G, Dong M, Fan X, He X (2021). New understanding of electrical activity brought by surface potential of cardiomyocytes. Sci. Rep..

[3] Tan J, Zhang B, Luo Y, Ye S (2017). Ultrafast vibrational dynamics of membrane-bound peptides at lipid bilayer/water interface. Angew. Chem. Int. Ed..

[4] Moleón JA, Moya AA (2009). Transient electrical response of ion-exchange membranes with fixed-charge due to ion adsorption: A network simulation approach. J. Electroanal. Chem..

[5] Rosen MR (1985). Cellular electrophysiology of digitalis toxicity. J. Am. Coll. Cardiol..

[6] Peluffo RD, Berlin JR (2012). Membrane potential-dependent inhibition of the Na^+^, K^+^-ATPase by para-nitrobenzyltriethylammonium bromide. Mol. Pharmacol..

[7] Arispe N, Diaz JC, Simakova O, Pollard HB (2008). Heart failure drug digitoxin induces calcium uptake into cells by formingtrans membrane calcium channels. PNAS USA.

[8] Haucka C (2009). Isoform specificity of cardiac glycosides binding to human Na^+^, K^+^-ATPase α1β1, α2β1 and α3β1. Eur. J. Pharmacol..

[9] Martonosi AN (1985). The Enzymes of Biological Membranes.

[10] Flanagan RJ, Jones AL (2004). Fab antibody fragments: Some applications in clinical toxicology. Drug Saf..

[11] Botelho AFM, Pierezan F, Soto-Blanco B, Melo MM (2019). A review of cardiac glycosides: Structure, toxicokinetics, clinical signs, diagnosis and antineoplastic potential. Toxicon.

[12] Fozzard HA, Haber E, Jennings RB, Katz AM, Morgan HE (1991). The Heart and Cardiovascular System: Scientific Foundations.

[13] Ross MH, Pawlina W (2010). Histology: A Text and Atlas, with Correlated Cell and Molecular Biology.

[14] Su J, Li C, Su Z (1999). Heart from Basic to Clinical.

[15] Delgado AV, González-Caballero F, Hunter RJ, Koopal LK, Lyklema J (2007). Measurement and interpretation of electrokinetic phenomena. J. Colloid Interface Sci..

[16] Xiao J (2021). Regulation and drug modulation of a voltage-gated sodium channel: Pivotal role of the S4–S5 linker in activation and slow inactivation. Proc. Natl. Acad. Sci. USA..

[17] Tseng S, Hsieh TH, Yeh LH, Wang N, Hsu JP (2013). Electrophoresis of a charge-regulated soft sphere: Importance of effective membrane charge. Colloids Surf. B..

[18] Zhou ZK, Gu TR, Ma JM (1996). Colloid Chemistry Basis.

[19] Matsui H, Schwartz A (1968). Mechanism of cardiac glycoside inhibition of the Na, K-dependent ATPase from cardiac tissue. Biochim. Biophys. Acta..

[20] Ling GN, Fu YZ (1987). An electronic mechanism in the actions of drugs and other cardinal adsorbents. I. Effects of ouabain on the relative affinities of the cell surface beta- and gamma-carboxyl groups for K+, Na+, glycine and other ions. Physiol. Chem. Phys. Med. NMR..

[21] Moraes C, Sun Y, Simmons CA (2011). (Micro)managing the mechanical microenvironment. Integr. Biol..

[22] Anandhi D, Pandit VR, Kadhiravan T, Soundaravally R, Raju KP (2019). Cardiac arrhythmias, electrolyte abnormalities and serum cardiac glycoside concentrations in yellow oleander (Cascabelathevetia) poisoning: A prospective study. Clin. Toxicol..

[23] Miura DS, Rosen MR (1978). The effects of ouabain on the transmembrane potentials and intracellular potassium of canine cardiac Purkinje fibers. Circ. Res..

[24] Payandeh J, Scheuer T, Zheng T, Catterall WA (2011). The crystal structure of a voltage-gated sodium channel. Nature.

[25] Bartos DC, Grandi E, Ripplinger CM (2015). Ion channels in the heart. Compr. Physiol..

[26] Wisedchaisri G (2019). Resting-state structure and gating mechanism of a voltage-gated sodium channel. Cell.

[27] Armstrong CM, Bezanilla F (1973). Currents related to movement of the gating particles of the sodium channels. Nature.

[28] Lenaeus MJ (2017). Structures of closed and open states of a voltage-gated sodium channel. PNAS USA.

[29] Pugsley MK, Curtis MJ, Hayes ES (2015). Biophysics and molecular biology of cardiac ion channels for the safety pharmacologist. Handb. Exp. Pharmacol..

[30] Yu FH, Catterall WA (2003). Overview of the voltage-gated sodium channel family. Genome Biol..

[31] Hu ZS (2011). Negative charge of residue E375 in the P-Loop of sodium channel Nav1.5 determines channel conductance and steady-state inactivation. J. Biophys..

[32] Gamal El-Din TM, Lenaeus MJ, Zheng N, Catterall WA (2018). Fenestrations control resting-state block of a voltage-gated sodium channel. PNAS USA.

[33] Ledwitch KV, Robertsm AG (2017). Cardiovascular ion channel inhibitor drug-drug interactions with P-glycoprotein. AAPS J..

[34] Ng GA (2017). Feasibility of selection of antiarrhythmic drug treatment on the basis of arrhythmogenic mechanism: Relevance of electrical restitution, wavebreak and rotors. Pharmacol. Ther..

[35] Ahmed M, JalilyHasani H, Ganesan A, Houghton M, Barakat K (2017). Modeling the human nav1.5 sodium channel: Structural and mechanistic insights of ion permeation and drug blockade. Drug Des. Dev. Ther..

[36] Ye S, Li H, Wei F, Jasensky J, Boughton AP, Yang P, Chen Z (2012). Observing a model ion channel gating action in model cell membranes in real time in situ: membrane potential change induced alamethicin orientation change. J. Am. Chem. Soc..

[37] Tamagawa H, Funatani M, Ikeda K (2016). Ling’s adsorption theory as a mechanism of membrane potential generation observed in both living and nonliving systems. Membranes.

[38] Lee CH (2014). NMDA receptor structures reveal subunit arrangement and pore architecture. Nature.

[39] Kefauver JM, Ward AB, Patapoutian A (2020). Discoveries in structure and physiology of mechanically activated ion channels. Nature.

[40] Shipston MJ (2014). Ion channel regulation by protein S-acylation. J. Gen. Physiol..

[41] Shen H (2018). Structural basis for the modulation of voltage-gated sodium channels by animal toxins. Science.

[42] Soler F, Fernandez-Belda F, Gomez-Fernandez JC (1992). The Ca^2+^ release channel in junctional sarcoplasmic reticulum: gating and blockade by cations. Int. J. Biochem..

[43] Smith TW (1988). Digitalis: Mechanisms of action and clinical use. N. Engl. J. Med..

[44] Gettes LS (1992). Electrolyte abnormalities underlying lethal and ventricular arrhythmias. Circulation.

[45] Hoppe LK (2018). Association of abnormal serum potassium levels with arrhythmias and cardiovascular mortality: A systematic review and meta-analysis of Observational studies. Cardiovasc. Drugs Ther..

[46] Maruyama Y, Chambers DJ, Ochi M (2013). Future perspective of cardioplegic protection in cardiac surgery. J. Nippon Med. Sch..

[47] Kazanskaia GM, Volkov AM, D’iakonitsa TM, Zhdanov GP (2005). Endothelium of myocardium microvessel under conditions of hypothermia, ischemia, reperfusion and pharmaco-cold cardioplegia with calcium antagonist. Tsitologiia.

[48] Zhou YY (2000). Culture and adenoviral infection of adult mouse cardiac myocytes: Methods for cellular genetic physiology. Am. J. Physiol. Heart Circ. Physiol..

[49] McGarry SJ, Williams AJ (1993). Digoxin activates sarcoplasmic reticulum Ca^2+^-release channels: A possible role in cardiac inotropy. Br. J. Pharmacol..

[50] Schulz M, Schmoldt A (2003). Therapeutic and toxic blood concentrations of more than 800 drugs and other xenobiotics. Pharmazie..

