# Supplementary material for: Relationship among surface electric double layer of cardiomyocyte membrane and toxicology of digoxin and opening of ion channels
Source: Sci Rep. 2022 Dec 1;12:20749. doi: 10.1038/s41598-022-25205-2 (PMC9715572; doi:10.1038/s41598-022-25205-2)
Supplement: Supplementary file 1 — Supplementary Figures. [file 41598_2022_25205_MOESM1_ESM.pdf]

Relationship among surface electric double layer of cardiomyocyte membrane and toxicology of digoxin and opening of ion channels

Ying Zhou*, Yanfei Hao, Pei Sun, Ming Chen, Ting Zhang, Hong Wu

Supplementary Figures

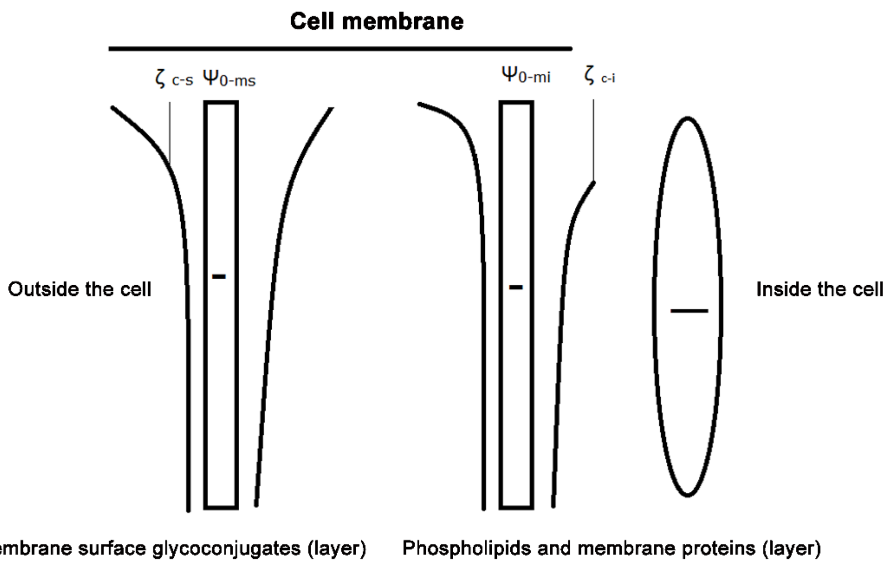

Supplementary Fig. 1 │ Surface potential distribution of cardiomyocytes in resting state

Ψ_{ms} is the electric potential of the surface glycoconjugates on the cardiomyocyte membrane, and Ψ_{mi} is the electric potential of the phospholipid and membrane protein layer inside the membrane, which includes the influence of the transmembrane potential. ζ_{ms} and ζ_{ci} are the electromotive potential on the surface of the cardiomyocyte membrane and in the cell, respectively.

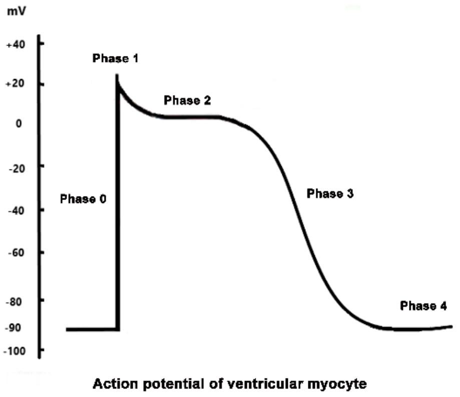

Supplementary Fig. 2 │ Brief introduction of each phase of action potential of ventricular myocytes

Phase 0: rapid depolarization; Phase 1: early rapid repolarization; Phase 2: plateau phase; Phase 3: terminal rapid repolarization; Phase 4: return to resting membrane potential.

Among them, the change trend of the action potential depends on the two parallel electric double layer structures on the cell surface: 1. The overshoot of Phase 0 is related to the separation of the electric double layer inside the cell membrane at this stage; 2. The plateau phase is the total electrochemical potential equilibrium inside and outside the cell, and two parallel electric double layers are its structural basis; 3. Phase 4 ion exchange. Ion exchange is the dynamic equilibrium of local single ions, which is completed in the adsorption layers on both sides of the plasma membrane (i.e., Ψ_{mi} and the adsorption layer it belongs to).

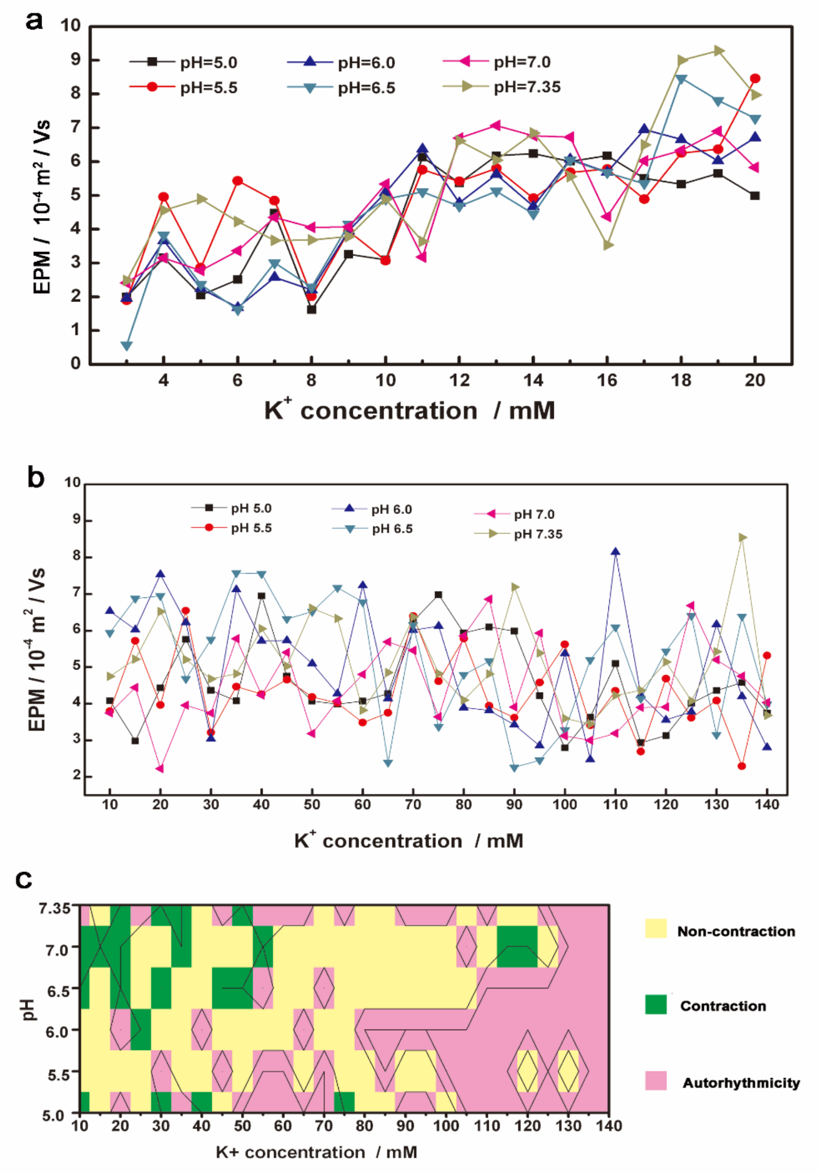

Supplementary Fig. 3 │ The relationship among K^+ concentration, cardiomyocyte EPM and behavior.

Constant ionic concentration at 153 mM was composed of 2 mM $CaCl_2$, 10-147 mM NaCl, 3-140 mM KCl, 5mM glucose, 20 mM HEPES and 0.57 mM MPHC (pH 5.0-7.35). (when the K^+ concentration is 3-140 mM, the theoretical value of the transmembrane potential is about -96.8 – 0 mV). The trend of cardiomyocyte EPM at 3-20 mM K^+ concentration (a), the trend of cardiomyocyte EPM at 10-140 mM K^+ concentration (b); behavior of cardiomyocytes at K^+ concentrations of 10-140 mM. Behaviors, i.e., non-contraction (with or without external electric field); contraction (After applying an external electric field); autorhythmiclty (without external electric field) (c).
